# Production of Pectic Oligosaccharides from Citrus Peel via Steam Explosion

**DOI:** 10.3390/foods13233738

**Published:** 2024-11-22

**Authors:** Toni-Ann Martorano, Kyle L. Ferguson, Randall G. Cameron, Wei Zhao, Arland T. Hotchkiss, Hoa K. Chau, Christina Dorado

**Affiliations:** 1 U.S. Horticultural Research Laboratory, Agricultural Research Service, United States Department of Agriculture, Fort Pierce, FL 34945, USA; 2 Eastern Regional Research Center, Agricultural Research Service, United States Department of Agriculture, Springfield, PA 19038, USA

**Keywords:** pectin, polysaccharide, pectic oligosaccharide, citrus, steam explosion, valorization, value added

## Abstract

Steam explosion (STEX) of peel from commercially juice-extracted oranges was used to convert peel pectin into pectic oligosaccharides (POSs). Surprisingly uniform populations, based on the polydispersity index (PDI; weight-average molecular weight (M_w_)/number-average molecular weight (M_n_)) of POSs, were obtained from the Hamlin and Valencia varieties of *Citrus sinensis*. The POSs from Hamlin and Valencia peel had PDI values of (1.23 ± 0.01, 1.24 ± 0.1), respectively. The M_w_ values for these samples were 14.9 ± 0.2 kDa for Hamlin, and 14.5 ± 0.1 kDa for Valencia, respectively. The degree of methyl-esterification (DM) was 69.64 ± 3.18 for Hamlin and 65.51 ± 1.61 for Valencia. The composition of the recovered POSs was dominated by galacturonic acid, ranging from 89.1% to 99.6% of the major pectic sugars. Only the Hamlin sample had a meaningful amount of rhamnose present, indicating the presence of an RG I domain. Even so, the Hamlin sample’s degree of branching (DBr) was very low (2.95).

## 1. Introduction

Pectin is a nearly ubiquitous polysaccharide found in plant primary cell walls and middle lamellae [1,2,3]. It is composed primarily of three major domains: homogalacturonan (HG), rhamnogalacturonan I (RG I), and rhamnogalacturonan II (RG II) [4,5,6,7]. HG is a linear homopolymer of a-1,4 linked D-galacturonic acid (GalA) with a degree of polymerization (number of contiguous GalA subunits) generally ranging between 80 and 120 GalAs [8]. A variable proportion of the GalA subunits may be methyl-esterified at the C6 position. The percentage of methyl-esterified GalAs is defined as the degree of methyl-esterification (DM). RG I has a backbone composed of a repeating dimer of GalA and rhamnose (Rha), with neutral sugar side branches of galactans, arabinans, or arabinogalactans off of Rha subunits. RG II is a relatively minor component, but functionally significant [7]. Other, less common, domains are apiogalacturonan and xylogalacturonan [9].

Pectin has biological functions that mirror many of its technological applications. In plant cells, pectin functions include cell–cell adhesion, structural support, hydration control, the positioning of leaf and floral primordia, and acting as signaling molecules in plant biochemical pathways [4,6,10]. Pérez et al. [11] described three-dimensional models of specific pectin structural domains, but a three-dimensional model of its global in vivo structure has not been obtained.

Commercially, pectin’s dominant use is in the food industry, where it is utilized for its functional properties related to gelation, thickening, stabilizing, and emulsification [12,13,14]. More recently, its applications have been expanding into other markets related to health [14]. In many of these markets, it is fragments of pectin (pectic oligosaccharides, or POSs, and modified citrus pectin) that have been reported to have biological activity or pharmaceutical properties [15,16,17,18,19]. The potential applications that have been investigated include uses as prebiotics [16,18,20,21,22,23,24,25,26], apoptosis induction of colon and prostate cancer cells [15,27,28], galectin-3 antagonist during cancer metastasis, fibrosis reduction and heavy metal detoxification [29], and the controlled release of pharmaceuticals [30,31], among others [32]. POSs have also been shown to act as drug delivery excipients [31], and as elicitors for plant defense responses [4,33,34], among others. Udchumpisai et al. [35] demonstrated that POSs can biostimulate rice seed growth activation and that the larger POSs have a positive effect on growth and metabolism.

A variety of methods have been reported for the production of POSs [16,17,18,19,20,21,22,23,24,25,26,36]. STEX was chosen because it can be operated as a continuous process and the pectin recovery is equivalent to the amount of pectin present in raw peel as determined by enzymatic digestion [37]. In this work, data are provided on the major pectic sugar composition of the recovered POSs produced via STEX and their macromolecular and structural properties, including weight fraction (%), weight-average molecular weight (Mw), the calculated poly-dispersity index (PDI, *M_w_*/*M_n_*), intrinsic viscosity (*η*), radius of gyration, Mark–Houwink–Sakurada exponent values (a), degree of polymerization (DP), degree of methyl-esterification (DM), and degree of branching in RG I (DBr).

## 2. Materials and Methods

### 2.1. Static Steam Explosion

Fresh, juice-extracted orange peel (*Citrus sinensis* var. Hamlin and Valencia) was obtained from a local juice processor in Ft. Pierce, FL, USA. Juice extraction was accomplished using Brown International Juice Extractors (Winter Haven, FL, USA); consequently, the peel was obtained as halved fruit. Hamlin peel was obtained on 24 January 2022, and Valencia peel was obtained on 10 March 2022 (Valencia 1) and 26 April 2022 (Valencia 2). The initial size reduction was accomplished using two passes through the blades of a Fitz-Mill fitted with no screen (Model D-S6, Fitzpatrick Company, Westwood, MA, USA). Further size reduction was completed using a Robot-Coupe (Model R 23, Robot-Coupe, S.N.C., Vincennes Cedex, France) food processor for 15 s. A total of 500 g of the size-reduced peel was added to a clean bucket containing 5 L of deionized water. This slurry was added to the clean Robot-Coupe for an additional 15 s homogenization. Approximately 600 g of this mixture was weighed into a beaker and brought to a pH of 2 using 6 N HCl. It was then placed into the vertical pipe of a static steam explosion system (Figure S1) and held at 140 °C for 30 min (~50 psi). After pressure release, the sample was collected and stored at 4 °C until pectin recovery took place, no longer than 2 h.

### 2.2. Pectin Recovery

The sample cooled after the steam explosion was vacuum-filtered (Glass Microfiber Filter (125 mm diameter, Whatman GF/F, GE Healthcare, Life Sciences, Malbrough, MA, USA) and the filtrate pH was adjusted to 2 using 6 N HCl. The solution was then precipitated using 95% ethanol to bring the final concentration to 55%. The sample was precipitated overnight, and covered, at room temperature. The sample was then centrifuged at 12,000 rpm for 20 min (Model Avanti JE, Beckman Coulter, Inc. Brea, CA, USA). The pellet was washed with 57% ethanol and centrifuged again under the same conditions. The pellet was stored at −20 °C before it was lyophilized (FreeZone Freeze Dry System; Labconco, Kansas City, MO, USA), or stored at −20 °C until ready for lyophilization.

### 2.3. Compositional Analysis of Recovered Pectin

The compositional analysis of sugars (rhamnose, arabinose, galactose, glucose, xylose, fructose, sucrose, cellobiose, and galacturonic acid) was determined by hydrolysis of 2 mg of the freeze-dried recovered pectin sample diluted in 2 mL of DI water with 43.2 μL of two pectinases (DSM, PAC, Batch 16B04V1, pectinase activity, 49.43 U mL^−1^, and Rapidase PNS, pectinase activity, 58.29 U mL^−1^), 21.6 μL cellulase (Novozyme, Cellic CTec2, VCPI0003, cellulase activity, 208.21 FPU mL^−1^), and 21.6 μL β-glucosidase (Novozyme 188, DCN00205, β-glucosidase activity, 270.67 U mL^−1^) enzymes with rotation for 24 h at 45 °C. To prevent microbial growth, 6 μL of cycloheximide (10 mg ml^−1^ stock) were added. Samples were then filtered, using a 0.45 μm GD/X Nylon syringe filter (Whatman, Cytiva, Marlborough, MA, USA) to remove insoluble solids prior to analysis. Sugars were quantified and identified by direct high-performance ion exchange chromatography (HPIEC) using a Dionex CarboPac PA-1 pellicular anion-exchange column (4 × 250 mm) and pre-column (4 × 50 mm) as described previously [38] with some modification. Specifically, the percent of each buffer used, time, and flow for the sugar analysis can be seen in Table S1. The waveform method and temperature settings were input manually, using the Antec Decade Elite system digital display. Data collection and analysis were completed using Agilent OpenLab CDS Chemstation Rev C. The samples were analyzed in triplicate.

### 2.4. Macromolecular Characterization of Recovered Pectin

Pectin (Hamlin and Valencia 1) samples were prepared in 0.05 M NaNO_3_/0.01% NaN_3_ and stirred overnight at room temperature, and a 0.45 μm filter was used to filter the solutions (Millex-HV, PVDF, Millipore Corp., Billerica, MA, USA). The delivery system consisted of a model 1260 series pump, Infinity II degasser, multicolumn thermostat MCT, and auto-sampler (Agilent Technologies, Waldbronn, Germany). Two biocompatible inline solvent filter assemblies, PEEK/SS, 0.5 μm (Cole-Parmer, Burlington, NJ, USA), were placed before and after three size-exclusion columns (TSK GMPWxl, 7.8 × 300 mm, 13 μm particle size, Tosoh Bioscience, Tokyo, Japan). The injection volume was 100 μL and the flow rate was set at 0.7 mL/min. The column set was heated at 35 °C. The Dawn Ambient-D3 multi-angle laser light-scattering photometer (MALLS, Wyatt Technology, Santa Barbara, CA, USA), ViscoStar-V4 differential pressure viscometer (DPV), 1728-TREX differential refractive index (RI, Wyatt Technology, Santa Barbara, CA, USA), and a UV-1260 Infinity spectrophotometer (UV, Agilent Technologies, Waldbronn, Germany) were connected in series. The detectors were aligned with bovine serum albumen (Sigma-Aldrich, St Louis, MO, USA). A narrow-monodispersed Pullulan 50 standard was used to normalize the MALLS 18-angle detector (P-82, JM Science, Grand Island, NY, USA). Astra software (Ver. 8.1.1.12) was used for collecting and analyzing the chromatograms (Wyatt Technology, Santa Barbara, CA, USA). The refractive index increment (dn/dc) value of 0.132 was used. The samples were analyzed in triplicate.

The DM was determined as described by Cameron et al. [39] using a modified titration method found in the United States Pharmacopeia [40]. Four replicates from each sample were performed.

Pectin architecture was explored by using the major pectic sugar composition and DM values. GalA/Rha ratios and the degree of branching (DBr) [41] were calculated. The ratio of GalA to Rha was calculated as a hypothetical representation of the HG/RG I ratio within the pectin samples [42,43]. DBr was calculated as the ratio of Gal + Ara/Rha. DM was used to calculate the average MW of a GalA within the HG domain using the formulas below, where 18.00 Daltons are lost with the water loss in condensation and 32.04 Daltons are added for the addition of a methyl-ester. Estimating the average MW of a GalA allowed us to estimate the DP of the HG domains in the POS from each sample based on the estimated M_w_.

(1) $$\;\;\;\;\;GalA-H_{2}O=194.14-18.00=176.14$$

(2) $$GalAMe-H_{2}O=\left(176.14+32.04\right)-18.00=190.18$$

(3) $$Ave\;GalA-H_{2}O\;MW=GalA-H_{2}O\times (\left(100-DM\right)÷100)$$

(4) $$Ave\;GalAMe-H_{2}O\;MW=GalAMe-H_{2}O\times (DM÷100)$$

(5) $$GalA\;Ave\;MW=Ave\;GalA-H_{2}O\;MW+Ave\;GalAMe-H_{2}O\;MW$$

### 2.5. Data Analysis

Statistical analyses were performed using the one-way ANOVA (*p* < 0.05) function of Microsoft Excel (version 2304) and Tukey’s HSD (honestly significant difference) test at a confidence level of *p* < 0.05 was performed for multiple comparisons using JMP Pro Statistical Software Version 16 (SAS Institute Inc., Cary, NC, USA).

## 3. Results and Discussion

### 3.1. Pectin Yield and Sugar Composition

The highest pectin recovery was seen with the Hamlin sample, and the lowest was in the Valencia 2 sample, while Valencia 1 pectin recovery was similar to Hamlin (Table 1). Average temperatures and pressures within the STEX vessel were very similar in each run. The pectin recovery was similar to the injected steam extraction of Valencia orange pectin for 6 min in pH 2 HCl at 120 °C, 15 psi [44].

The pectin sugar composition (Table 2) indicated that GalA was the dominant sugar in each sample, ranging from 89.15% in the Hamlin sample to 99.61 and 96.59 in Valencia 1 and Valencia 2, respectively. Only the Hamlin sample appeared to contain any RG I component, with a GalA/Rha ratio of 20.36 and a DBr of 2.95. For Valencia 1, the GalA/Rha ratio was 190.79, with a minimal DBr of 0.51, and no Gal was detected. No Rha was detected in the Valencia 2 sample although minimal amounts of Gal and Ara were detected. Since it is possible that the sugar composition analysis enzymes did not hydrolyze the rhamnogalacturonan I, we repeated the monosaccharide analysis using methanolysis [21] and found similar results (Hamlin: 82.57% GalA, 7.59% Xyl, 4.76% Glc, 2.38% Gal, 1.22% GlcA, 1.08% Ara, 0.37% Rha; Valencia 1: 99.1% GalA, 0.23% Xyl, 0.21% Ara, 0.18% Gal, 0.15% GlcA, 0.07% Rha, 0.07% Glc); Valencia 2: 98.08% GalA, 0.74% Ara, 0.62% Gal, 0.17% GlcA, 0.16% Xyl, 0.15% Rha, 0.07% Glc. Therefore, the STEX process produced a homogalacturonan structure by preferentially hydrolyzing the rhamnogalacturonan I domains of pectin that are known to be less acid-resistant [45]. The combination of homogenization and steam explosion may have been similar to high-pressure homogenization that depolymerized citrus pectin [46] and increased the uronic acid content of carrot pectin [47]. STEX POSs have similar sugar composition to POS1 and modified citrus pectin, which are reported to have anti-adhesive activity for Shiga toxin-producing *Eschericia coli* [25]. In previous reports of orange peel POSs with prebiotic properties, the POSs had high levels of arabinose and relatively lower levels of galacturonic acid compared to the STEX POSs [21,24,25].

(6) $$\%=\left[\frac{X}{Rha+Ara+Gal+GalA}\right]*100,\;\;\;\;Where\;X=Rha,\;Ara,\;Gal,\;or\;GalA$$

### 3.2. Macromolecular Characterization and Architecture

**Figure 1:**
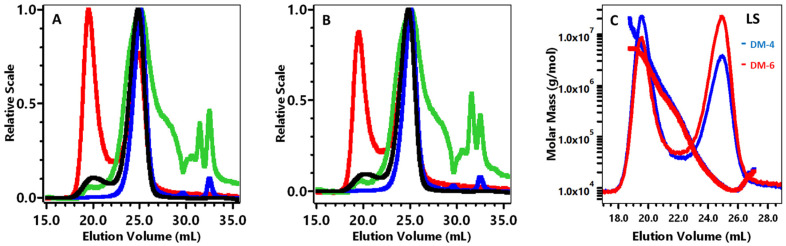
High-performance size-exclusion chromatography analysis of the Hamlin (DM-D4) (**A**) and Valencia (DM-D6) (**B**) varieties; superimposed calibration curve of Hamlin and Valencia (DM-D4, DM-D6) (**C**). In Figure 1A,B, HPSEC detectors were light scattering at 90 °C (-), differential pressure viscometer (-), refractive index (-), and ultraviolet absorption at 280 nm (-). In Figure 1C, HPSEC detectors were light scattering at 90 °C, (DM-D4) and HPSEC detectors were light scattering at 90 °C, DM-D6.

Representative HPSEC-MALLS chromatograms for each sample demonstrated a bimodal POS peak (Figure 1A,B, light scattering), with peak 1 eluting 18.7–22.1 mL, which represented 1% of an integrated peak area of the sample, and the major peak 2 eluting 22.1–26.6 mL, which represented 99% of the sample for both Hamlin and Valencia 1 (Table 3).

The polydispersity index is defined as *M_w_*/*M_n_* and used to determine the broadness of molecular weight distribution as demonstrated in Table 3. The total chromatogram (TC) including all peak areas 18.7–26.6 mL, had an *M_w_*/*M_n_* of 2.52 for Hamlin and 2.14 for Valencia 1. Compared to peak 2, the peak 1 polydispersity was much broader, the molar mass (*M_w_*) was much higher, and the Mark–Houwink constant (*a*) was more compact, which indicated pectin aggregation. In contrast, the peak 2 *M_w_* and intrinsic viscosity (*η*) for both samples were the same (Table 3, Figure 1C). The peak 2 14 kDa *M_w_* was much lower than the 367–414 kDa *M_w_* for orange pectin that was acid extracted by other methods [22,28], which indicated that depolymerization occurred during STEX. The radius of gyration (*Rg*) (Table 3) and UV chromatograms (Figure 1A,B) for both samples were similar. A comprehensive study described the conformation of polysaccharides with the Mark–Houwink–Sakurada exponent [48,49]. The M-H exponent (*a*) for Hamlin and Valencia 1 in this experiment indicated a random coil shape molecule. Pectin with a random coil shape was reported to bind to anthocyanins with anti-oxidant properties [50]. Hellin et al. [51] used commercial lime pectin de-esterified by 0.1 M NaOH at 4 °C and hydrolyzed with 0.1 M HCL at 80 °C for 72 h, that revealed a bimodal light-scattering peak with the first peak reported as aggregates. The main peak 2 was reported to possess values of 18.5 kDa *M_w_* and 0.75 dL/g *η*, which were higher than the Hamlin and Valencia STEX peak 2 values. However, their α was 0.87, which agreed with the random coil shape that we observed for Hamlin and Valencia STEX peak 2.

Singh and Tingirikari [26] reported a wide range of sizes for POSs, from a degree of polymerization (DP) of 1–10, with *M_w_* up to 4000 kDa, depending on the production method. Cano et al. [24] also reported a wide range of *M_w_* ranging from 1200 to 3600 kDa obtained from citrus fruit peel. Much smaller POSs were reported by Di et al. [25], with *M_w_* ranging between 9.2 and 811 kDa. The arabinose-rich POS was the most bifidogenic, while a lower molecular weight and de-methyl-esterification enhanced anti-adhesive activity against *E. coli* O157:H7 binding to human HT29 cells [25].

Single-factor ANOVA of *M_n_*, *M_w_*, polydispersity, and (*η*) (Table 3) demonstrated significant differences between the means (*F* = 553, *p*-value = 1.57^−7^; *F* = 456.8, *p*-value = 2.77^−7^; *F* = 300.45, *p*-value = 9.66^−7^, and *F* = 12,757.5, *p*-value = 1.30^−11^, respectively). All samples could be considered high-DM pectins (>50%; Table 4). No significant difference was found for the DMs of the samples (Table 4, *F* = 1.05, *p*-value = 0.3893). Based on the estimated DM, *M_w_*, and the calculations from Formulas (1)–(5) we estimated the degree of polymerization (DP) for each of the POSs. Hamlin was the largest with a DP of 51.8. Valencia 1 had a DP of 33.1, and Valencia 2 had a DP of 27.7. Since the POSs are mainly homogalacturonan, they are oligogalacturonic acids with DPs of up to 50 galacturonic acid residues detected with HPAEC-PAD [52]. The HPAEC-PAD method can also resolve individual malto-oligosaccharides up to 70 glucose residues [53], which expands the traditional oligosaccharide definition beyond DP 10. Therefore, our POS DP calculations appear to be reasonable.

## 4. Conclusions

Steam explosion of commercial orange peel from Hamlin and Valencia oranges produced pectic oligosaccharides and polysaccharides with homogalacturonan composition, high degree of esterification, low molar mass, and a random coil shape. Acidic steam explosion extraction provides a rapid, continuous method that preferentially hydrolyzes the pectin rhamnogalacturonan I domain during POS preparation. The application of these pectic oligosaccharides remains to be investigated, but their structure and composition suggests that they may be able to prevent the adhesion of pathogenic Shiga toxin-producing *Eschericia coli* and bind anthocyanins with anti-oxidant properties. Orange peel POSs represent a potentially valuable co-product of orange juice manufacturing with functional food properties.

## Figures and Tables

**Table 1:** Pectin yield obtained from STEX of Hamlin and Valencia juice-extracted orange peel.

	Date	Start Material (g)	Average Temperature (°C)	Average Pressure (PSI)	Pectin Extracted (mg)	Percent Yield (%)
**Hamlin**	1/24/22	601	140.9	41.7	298.5	16.05
**Valencia 1**	3/10/22	602	141.3	42.3	272.8	14.56
**Valencia 2**	4/26/22	598	142.0	44.3	171.1	9.23

**Table 2:** The average (Mean) and standard error (SE) of the major pectic sugars given in percent dry weight (% dw) and descriptors of pectin architecture (GalA/Rha, DBr (GalA + Ara/Rha)). Percent GalA (%GalA) was the percentage of the total sample that was GalA. The percentage of the major pectin sugars (%) was calculated using Equation (6). ND = Not Detected.

	Hamlin			Valencia 1			Valencia 2		
	Mean	SE	%	Mean	SE	%	Mean	SE	%
**Rha**	0.0014	0.0004	2.1898	0.0002	0.0003	0.2604	ND		
**Ara**	0.0004	0.0001	0.6125	0.0001	0.0000	0.1322	0.0003	0.0000	0.368526
**Gal**	0.0036	0.0003	5.8578	ND			0.0027	0.0006	3.040671
**GalA**	0.0564	0.0011	91.3399	0.0844	0.0059	99.6074	0.0848	0.0045	96.5908
**GalA/Rha**	20.3560			190.7948					
**DBr (Gal + Ara/Rha)**	2.9548			0.5079					
**%GalA**	89.1502			99.6074			96.5908		

**Table 3:** The polydispersity (*M_w_*/*M_n_*), weight-average molecular weight (*M_w_*), intrinsic viscosity (*η*), radius of gyration (*R_gz_*), and Mark–Houwink–Sakurada exponent (a) of Hamlin and Valencia as studied by HPSEC. The total area values are the average of a triplicate set of RI measurements ± standard deviations.

Sample	Integrated Peak Range	Weight Fraction% ^2^	*M_w_*/*M_n_*	*M_w_* × 10^−3^	*η_w_* (dL/g)	*R_gz_* (nm)	M-H (a)
Hamlin							
TC ^1^	18.7–26.6	100	2.52 ± 0.02	30.7 ± 0.1	0.45 ± 0.001	24.0 ± 2	0.819 ± 0.01
Peak 1	18.7–22.1	1.0 ± 0.1	2.99 ± 0.3	1656 ± 160	6.1 ± 0. 4	30.0 ± 1	0.586 ± 0.04
Peak 2	22.1–26.6	99 ± 0.1	1.23 ± 0.01	14.9 ± 0.2	0.39 ± 0.001	ND	0.865 ± 0.01
Valencia 1							
TC^1^	18.7–26.6	100	2.14 ± 0.03	25.4 ± 0.5	0.44 ± 0.002	23.0 ± 2	0.843 ± 0.05
Peak 1	18.7–22.1	0.90 ± 0.1	2.22 ± 0.08	1264 ± 196	5.9 ± 0. 8	30.0 ± 2	0.520 ± 0.01
Peak 2	22.1–26.6	99± 0.1	1.24 ± 0.01	14.5 ± 0.1	0.40 ± 0.01	ND	0.887 ± 0.03

^1^ TC = Total chromatogram. ^2^ Percentage (%) of an integrated peak area over the total of all eluted peak areas.

**Table 4:** Degree of methyl-esterification (DM) of extracted pectins. SD = Standard deviation, SE = standard error of the mean. Averages with the same superscript are not significantly different.

Sample	Average	SD	SE
Hamlin	69.64 ^a^	6.360306	3.180153
Valencia V1	65.51 ^a^	3.212808	1.606404
Valencia V2	65.11 ^a^	4.59405	2.297025

## Data Availability

The original contributions presented in this study are included in the article/Supplementary Material; further inquiries can be directed to the corresponding author.

## Supplementary Materials

The following supporting information can be downloaded at: https://www.mdpi.com/article/10.3390/foods13233738/s1, Figure S1. Static steam explosion system; Table S1. HPLC method time, buffer concentration and flow.

## Note

The U.S. Department of Agriculture prohibits discrimination in all its programs and activities on the basis of race, color, national origin, age, disability, and where applicable, sex, marital status, familial status, parental status, religion, sexual orientation, genetic information, political beliefs, reprisal, or because all or part of an individual’s income is derived from any public assistance program. (Not all prohibited bases apply to all programs.) Persons with disabilities who require alternative means for communication of program information (Braille, large print, audiotape, etc.) should contact USDA’s TARGET Center at (202) 720-2600 (voice and TDD). To file a complaint of discrimination, write to USDA, Director, Office of Civil Rights, 1400 Independence Avenue, S.W., Washington, D.C. 20250-9410, or call (800) 795-3272 (voice) or (202) 720-6382 (TDD). USDA is an equal opportunity provider and employer.
