# Supplementary material for: Production of Pectic Oligosaccharides from Citrus Peel via Steam Explosion
Source: Foods. 2024 Nov 22;13(23):3738. doi: 10.3390/foods13233738 (PMC11640180; doi:10.3390/foods13233738)
Supplement: Supplementary file 1 [file foods-13-03738-s001.zip › foods-3259116-supplementary.pdf]

Table S1. Pectin yield obtained from STEX of Hamlin and Valencia juice-extracted orange peel.

	Date	Start Material (g)	Average Temperature (°C)	Average Pressure (PSI)	Pectin Extracted (mg)	Percent Yield (%)
**Hamlin**	1/24/22	601	140.9	41.7	298.5	16.05
**Valencia 1**	3/10/22	602	141.3	42.3	272.8	14.56
**Valencia 2**	4/26/22	598	142.0	44.3	171.1	9.23

Table S2. The average (Mean) and standard error (SE) of the major pectic sugars given in percent dry weight (% dw) and descriptors of pectin architecture (GalA/Rha, DBr (GalA + Ara/Rha)). Percent GalA (%GalA) was the percentage of the total sample that was GalA. The percentage of the major pectin sugars (%) was calculated using Equation (6). ND = Not Detected.

	**Hamlin**			**Valencia 1**			**Valencia 2**		
	**Mean**	**SE**	**%**	**Mean**	**SE**	**%**	**Mean**	**SE**	**%**
**Rha**	0.0014	0.0004	2.1898	0.0002	0.0003	0.2604	ND		
**Ara**	0.0004	0.0001	0.6125	0.0001	0.0000	0.1322	0.0003	0.0000	0.368526
**Gal**	0.0036	0.0003	5.8578	ND			0.0027	0.0006	3.040671
**GalA**	0.0564	0.0011	91.3399	0.0844	0.0059	99.6074	0.0848	0.0045	96.5908
**GalA/Rha**		20.3560			190.7948				
**DBr (Gal+Ara/Rha)**		2.9548			0.5079				
**%GalA**		89.1502			99.6074			96.5908	

Table S3. The polydispersity (M_w/M_n), weight-average molecular weight (M_w), intrinsic viscosity (η), radius of gyration (R_{gz}), and Mark–Houwink–Sakurada exponent (α) of Hamlin and Valencia as studied by HPSEC. The total area values are the average of a triplicate set of RI measurements. Standard deviations from triplicate analyses are given.

Sample	Integrated Peak Range	Weight Fraction %[2]	M_w/M_n	M_w×10^{-3}	η_w (dL/g)	R_{gz} (nm)	M-H (a)
~~DM-D4~~Hamlin							
TC[1]	18.7-26.6	100	2.52 ± 0.02	30.7 ± 0.1	0.45 ± 0.001	24.0 ± 2	0.819 ± 0.01
Peak 1	18.7-22.1	1.0 ± 0.1	2.99 ± 0.3	1656 ± 160	6.1 ± 0. 4	30.0 ± 1	0.586 ± 0.04
Peak 2	22.1-26.6	99 ± 0.1	1.23 ± 0.01	14.9 ± 0.2	0.39 ± 0.001	ND	0.865 ± 0.01
~~DM-D6~~Valencia 1							
TC[1]	18.7-26.6	100	2.14 ± 0.03	25.4 ± 0.5	0.44 ± 0.002	23.0 ± 2	0.843 ± 0.05
Peak 1	18.7-22.1	0.90 ± 0.1	2.22 ± 0.08	1264 ± 196	5.9 ± 0. 8	30.0 ± 2	0.520 ± 0.01
Peak 2	22.1-26.6	99± 0.1	1.24 ± 0.01	14.5 ± 0.1	0.40 ± 0.01	ND	0.887 ± 0.03

[1]TC = Total area values are average of triplicate set of RI measurements. Standard deviations from triplicate analyses are given. [2]Percentage (%) of an integrated peak area over the total of all eluted peak areas.

Table S4. Degree of methyl-esterification (DM) of extracted pectins. SD = Standard deviation, SE = standard error of the mean. Averages with the same superscript are not significantly different.

Sample	Average	SD	SE
**Hamlin**	69.64[a]	6.360306	3.180153
**Valencia V1**	65.51[a]	3.212808	1.606404
**Valencia V2**	65.11[a]	4.59405	2.297025
